# Thermal CO Oxidation and Photocatalytic CO_2_ Reduction over Bare and M-Al_2_O_3_ (M = Co, Ni, Cu, Rh, Pd, Ag, Ir, Pt, and Au) Cotton-Like Nanosheets

**DOI:** 10.3390/nano11051278

**Published:** 2021-05-13

**Authors:** Hee Jung Yoon, Ju Hyun Yang, So Jeong Park, Youngku Sohn

**Affiliations:** 1 Department of Chemistry, Yeungnam University, Gyeongsan 38541, Korea; hohoya0102@naver.com; 2 Department of Chemistry, Chungnam National University, Daejeon 34134, Korea; mil03076@naver.com (J.H.Y.); jsjs5921@naver.com (S.J.P.); 3 Department of Chemical Engineering and Applied Chemistry, Chungnam National University, Daejeon 34134, Korea

**Keywords:** γ-Al_2_O_3_ nanosheets, transition metal-loading, CO oxidation, photocatalytic CO_2_ reduction, physicochemical properties, hydrogen production

## Abstract

Aluminum oxide (Al_2_O_3_) has abundantly been used as a catalyst, and its catalytic activity has been tailored by loading transition metals. Herein, γ-Al_2_O_3_ nanosheets were prepared by the solvothermal method, and transition metals (M = Co, Ni, Cu, Rh, Pd, Ag, Ir, Pt, and Au) were loaded onto the nanosheets. Big data sets of thermal CO oxidation and photocatalytic CO_2_ reduction activities were fully examined for the transition metal-loaded Al_2_O_3_ nanosheets. Their physicochemical properties were examined by scanning electron microscopy, high-resolution transmission electron microscopy, X-ray diffraction crystallography, and X-ray photoelectron spectroscopy. It was found that Rh, Pd, Ir, and Pt-loading showed a great enhancement in CO oxidation activity while other metals negated the activity of bare Al_2_O_3_ nanosheets. Rh-Al_2_O_3_ showed the lowest CO oxidation onset temperature of 172 °C, 201 °C lower than that of bare γ-Al_2_O_3_. CO_2_ reduction experiments were also performed to show that CO, CH_3_OH, and CH_4_ were common products. Ag-Al_2_O_3_ nanosheets showed the highest performances with yields of 237.3 ppm for CO, 36.3 ppm for CH_3_OH, and 30.9 ppm for CH_4_, 2.2×, 1.2×, and 1.6× enhancements, respectively, compared with those for bare Al_2_O_3_. Hydrogen production was found to be maximized to 20.7 ppm during CO_2_ reduction for Rh-loaded Al_2_O_3_. The present unique pre-screening test results provided very useful information for the selection of transition metals on Al_2_O_3_-based energy and environmental catalysts.

## 1. Introduction

Aluminum oxide (Al_2_O_3_) has extensively been used as a heterogeneous catalyst in diverse catalytic reactions of CO oxidation [1,2,3,4,5,6,7,8,9,10,11,12,13,14,15,16,17,18,19], CO_2_ reduction [20,21,22,23], CO_2_ methanation/hydrogenation [20,24,25,26,27], and preferential oxidation of CO [28,29]. The efforts to increase the catalytic activity of the metal oxide have been devoted to the modification of the metal surface by loading of transition metals in groups of 9 (Co, Rh, and Ir), 10 (Ni, Pd, and Cu), and 11 (Cu, Ag, and Au). The morphology of a metal oxide support has also been a key factor for the enhancement of the catalytic activity [30]. It was reported that the role of an overlayer metal becomes different when the support metal oxide is different [2]. The relative catalytic activities of overlayer metals also become different when the catalytic application areas are different for the application to CO oxidation using transition metal-loaded Al_2_O_3_ catalysts.

Chen et al. prepared Pt nanoparticles (NPs) on Al_2_O_3_ and observed 100% CO conversion at −20 °C [1]. For the extremely high activity compared with a commercial Pt/Al_2_O_3_, based on the experimental and the density functional theory (DFT) calculations, they proposed that CO was initially adsorbed on Pt(OH) kink sites and reacted with OH to release gaseous CO_2_. Afterward, OH was regenerated by activation of O_2_ on terrace sites. Lou and Liu studied CO oxidation of single Pt atoms dispersed on Fe_2_O_3_ (highly reducible), ZnO (reducible), and γ-Al_2_O_3_ (irreducible) supports, and observed that the catalytic activity was in the order of Pt/γ-Al_2_O_3_ < Pt/ZnO < Pt/Fe_2_O_3_ [2], where the highly reducible support showed the highest catalytic activity. Chen et al. tested Pt/Al_2_O_3_ for preferential oxidation (PROX) of CO in H_2_ [29]. They concluded that CO conversion and CO_2_ selectivity reached up to 100% in a wide range of −30 °C to 120 °C. The high performance was attributed to a combination of Pt(OH) and metallic Pt on the Al_2_O_3_ support. Therefore, the adsorption of CO and the activation of O_2_ were optimally tuned to maximize the performance. For monodispersed single Pt atoms on θ-Al_2_O_3_, Moses-DeBusk et al. found that the CO oxidation did not follow a conventional Langmuir-Hinshelwood mechanism [11]. The Pt atom was first oxygenated, and then CO was bound to form a carbonate (CO_3_), which dissociated to generate gaseous CO_2_ [11]. Yang et al. employed the DFT calculation to investigate the relative CO oxidation for single-atom catalysts of Ni/γ-Al_2_O_3_ and Pd/γ-Al_2_O_3_ [7]. They reported that Ni showed an unexpectedly higher CO oxidation activity than the Pd. Ananth et al. synthesized Ag_2_O/γ-Al_2_O_3_ and (Ag_2_O + RuO_2_)/γ-Al_2_O_3_ catalysts and tested the CO oxidation performances to show that the catalytic activity was increased by the addition of RuO_2_ [6]. Han et al. reported a high CO oxidation activity at 30 °C for NiO (≤1 nm) on mesoporous Al_2_O_3_ prepared using atomic layer deposition [16]. The deactivation was found to be lowered with increasing the pre-annealing temperature.

For the application of Al_2_O_3_ to CO_2_ reduction, Zhao et al. synthesized Au/Al_2_O_3_/TiO_2_ nanocomposites, where the atomic-layer Al_2_O_3_ was sandwiched between the two layers [21]. They tested the photocatalytic CO_2_ reduction activity and observed CO (major) and CH_4_ (minor) as products. It was concluded that the charge transfer and surface charge recombination were highly influenced by Al_2_O_3_ interlayer thickness. Therefore, the maximum photocatalytic activity (37 μmol/g of CO and 2 μmol/g of CH_4_) was obtained by achieving optimum Al_2_O_3_ thickness (5 Å). Kwak et al. performed a temperature-programmed CO_2_ reduction with H_2_ on Ru/Al_2_O_3_ catalysts and observed CO and CH_4_ formation yields with activation energies of 82 kJ/mol and 62 kJ/mol, respectively [20]. It was found that CO formation selectivity was increased with increasing Ru metal dispersion but decreased with increasing Ru clustering and concluded that CO was not an intermediate species for CH_4_ formation. Chein and Wang tested CO_2_ methanation activities using Ni/Al_2_O_3_, Ru/Al_2_O_3_, and Ru-Ni/Al_2_O_3_ catalysts [27] and found that the hybrid bimetallic Ru-Ni showed higher performance than the monometallic catalysts.

Although numerous detailed in-depth studies have been performed using transition metal-loaded Al_2_O_3_ catalysts, no systematic comparison studies have been reported among diverse (M = Co, Ni, Cu, Rh, Pd, Ag, Ir, Pt, and Au) transition metal-loaded Al_2_O_3_ catalysts prepared by the same synthesis method. Motivated by this, we synthesized transition metal-loaded Al_2_O_3_ nanosheets and evaluated thermal CO oxidation activity as well as photocatalytic CO_2_ reduction activity. Consequently, the roles of overlayer transition metals were comparatively investigated in two totally different application reactions. Thereby, the present pre-screening test results provided useful information on the quick-selection of catalysts for thermal CO oxidation and photocatalytic CO_2_ reduction.

## 2. Materials and Methods

### 2.1. Catalysts Synthesis Procedures

For the synthesis of Al precursor nanosheets, 1 mmol of aluminum nitrate nonahydrate (Al(NO_3_)_3_ 9H_2_O, 98%, Sigma-Aldrich, St. Louis, MO, USA), 0.008 g of polyethylene glycol (PEG, M_n_ = 4000, Sigma-Aldrich, St. Louis, MO, USA), and 20 μL of oleic acid (≥99%, Sigma-Aldrich, St. Louis, MO, USA) were fully dissolved by magnetic stirring in a mixed solvent of 10 mL of deionized water and 15 mL of ethanol (99.9%, Samchun Chem., Gyounggi, Korea) for 20 min. After that, the solution was transferred into a Teflon-lined stainless-steel autoclave reactor, which was then tightly capped for sealing. The tightly capped reactor was placed in an oven setting at 200 °C for 12 h. After the thermal reaction, the reactor was naturally cooled to room temperature, and the finally obtained white precipitates were collected by washing with deionized water and ethanol repeatedly by centrifugation at 3600 rpm. The collected wet powder was fully dried in an oven setting at 80 °C for 24 h. To obtain Al_2_O_3_ nanosheets, the dried powder sample was thermally annealed at 600 °C for 2 h.

For transition metal (M = Co, Ni, Cu, Rh, Pd, Ag, Ir, Pt, and Au) loadings, 50 mg of Al_2_O_3_ nanosheets were fully dispersed in 20 mL ethanol, followed by adding 2 mol% of metal ions. The chemicals for metal ions were cobalt(II) nitrate hexahydrate (≥98%, Sigma-Aldrich, St. Louis, MO, USA), nickel(II) nitrate hexahydrate (≥98.5%, Sigma-Aldrich, St. Louis, MO, USA), copper(II) nitrate trihydrate (99%, Daejung, Gyounggi, Korea), rhodium(III) chloride hydrate (≥99.9%, Sigma-Aldrich, St. Louis, MO, USA), palladium(II) chloride (99.8%, Sigma-Aldrich, St. Louis, MO, USA), silver nitrate solution (0.1 N, Samchun Pure Chem., Gyounggi, Korea), platinum (III) chloride (≥99.9%, Sigma-Aldrich, St. Louis, MO, USA), iridium (III) chloride hydrate (≥99.9%, Sigma-Aldrich, St. Louis, MO, USA), and gold(III) chloride trihydrate (≥99.9%, Sigma-Aldrich, St. Louis, MO, USA). After complete mixing, the solvent was slowly evaporated by gentle heating (50 °C) while stirring. The dried M-loaded Al_2_O_3_ nanosheets were, again, thermally annealed at 600 °C for 2 h.

### 2.2. Sample Characterization

The surface morphologies of the Al-precursor, Al_2_O_3_ nanosheets, and M-loaded Al_2_O_3_ nanosheets were examined using a scanning electron microscope (SEM, Hitachi S-4800, Hitach Ltd., Tokyo, Japan) at conditions of 10 kV and 10 mA. X-ray crystallographic diffraction patterns were recorded using a PANalytical X’Pert Pro MPD diffractometer (PANalytical, Almelo, Netherland) with Cu Kα radiation (40 kV and 30 mA). Transmission electron microscopic (TEM) and high-resolution TEM (HRTEM) were obtained for bare Al_2_O_3_ nanosheets and the selected Ni- and Rh-loaded Al_2_O_3_ nanosheets using an FEI Tecnai G2 F20 TEM (Hillsboro, OR, USA) operated at 300.0 kV. X-ray photoelectron spectra were taken using a Thermo-VG Scientific K-alpha^+^ spectrometer (Thermo VG Scientific, Waltham, MA, USA) with a hemispherical energy analyzer. Attenuated total reflection Fourier-transform infrared spectroscopy (FT-IR) was employed using a Nicolet iS 10 FT-IR spectrometer (Thermo Scientific Korea, Seoul, Korea). The Brunauer-Emmett-Teller (BET) surface areas were measured using a ChemBET TPR/TPD analyzer (Quantachrome Instruments Corp., Boynton Beach, FL, USA) equipped with a thermal conductivity detector.

### 2.3. Thermal CO Oxidation and Photocatalytic CO_2_ Reduction Experiments

For thermal CO oxidation reactions, 20 mg of a catalyst was initially loaded into a U-shape quartz tube. After that, the tube was positioned in a temperature-programmed furnace. The temperature heating rate was 20 °C/min, and the flowing gas was CO(1.0%)/O_2_(2.5%)/N_2_ at a flow rate of 40 mL/min. The gas products from the outlet of the tube were monitored using a quadrupole mass spectrometer (RGA200, Stanford Research Systems, Sunnyvale, CA, USA). After the first run to a maximum temperature of 500 °C, the sample cell was naturally cooled to a room temperature of 25 °C. After that, the second run was performed at room temperature.

For photocatalytic CO_2_ reduction experiments, 3 mg of a catalyst was fully dispersed on a quartz disc (an area of 15.9 cm^2^) and placed in a stainless-steel reactor (volume ~40 mL) with additional deionized water (20 μL) beside the disc. After that, the reactor was tightly closed with a quartz window (0.3 cm thick and 4.5 cm diameter) on top. Afterward, pure (99.999%) CO_2_ gas was fully flushed and filled with the gas. For the photocatalytic CO_2_ reduction test, the reactor with the quartz window was placed under UVC (200–280 nm) lamps (a power density of 5.94 mW/cm^2^) for 12 h. After the UVC irradiation time, 0.5 mL of gas was taken and injected into a YL 6500 gas chromatograph (GC, Young In Chromass Co., Ltd., Seoul, Korea). For the analysis of the CO, CH_3_OH, CH_4_, and H_2_ products, the GC system was equipped with HP-Plot Q-PT column (Agilent Technologies, Inc., Santa Clara, CA, USA), 40/60 Carboxen-1000 column (Sigma-Aldrich, St. Louis, MO, USA), a Ni catalyst methanizer assembly, a thermal conductivity detector, and a flame ionization detector.

## 3. Results and Discussion

**Figure 1:**
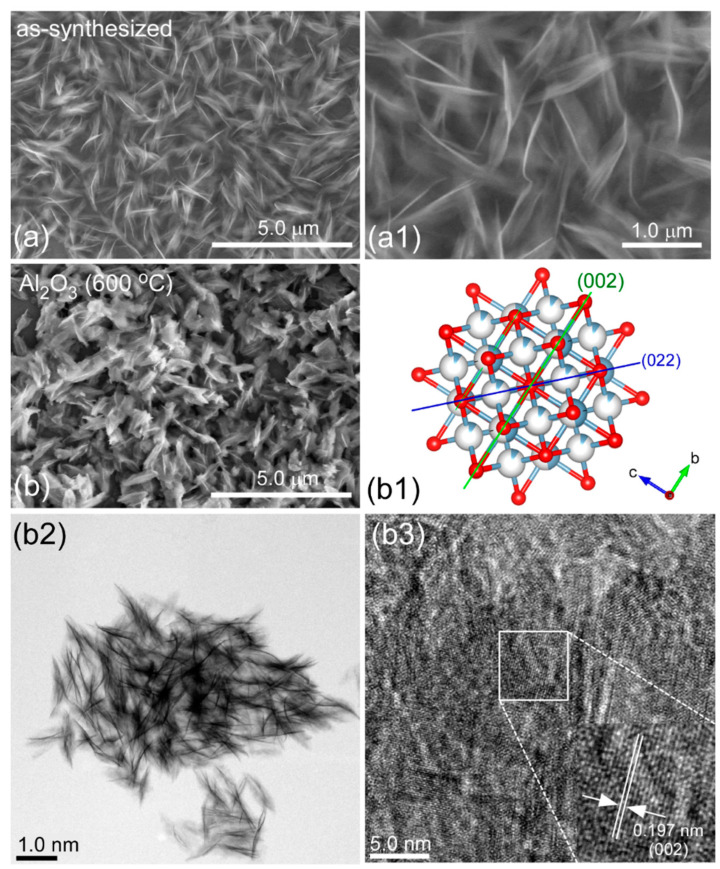
Scanning electron microscope (SEM) (**a**,**a1**,**b**), transmission electron microscope (TEM) (**b2**), high resolution TEM (**b3**) images of the as-synthesized Al-precursor (**a**,**a1**) and Al_2_O_3_ (**b**,**b2**,**b3**), and the structure projection (**b1**) of the (002) and (022) planes for cubic phase γ-Al_2_O_3_.

Figure 1(a,a1) show the SEM image of the as-prepared Al-precursor with a morphology of cotton-like nanostructures. Figure 1b shows the sample after thermal annealing at 600 °C, abbreviated as bare Al_2_O_3_. It appears that the morphology showed no significant change, but the nanosheets became somewhat compacted. The Brunauer-Emmett-Teller (BET) surface area of bare Al_2_O_3_ was measured to be 154.4 m^2^/g. The corresponding transmission electron microscope (TEM) image clearly showed the morphology of nanosheets. For the high-resolution TEM image of bare Al_2_O_3_, clear lattice fringes were seen, and the lattice spacing was estimated to be 0.197 nm. This was well-matched to the (002) crystal plane of cubic phase gamma-Al_2_O_3_. This was further discussed in detail below. The structure projection of the (002) and (022) planes for Al_2_O_3_ are shown in Figure 1(b1) for visual understanding.

**Figure 2:**
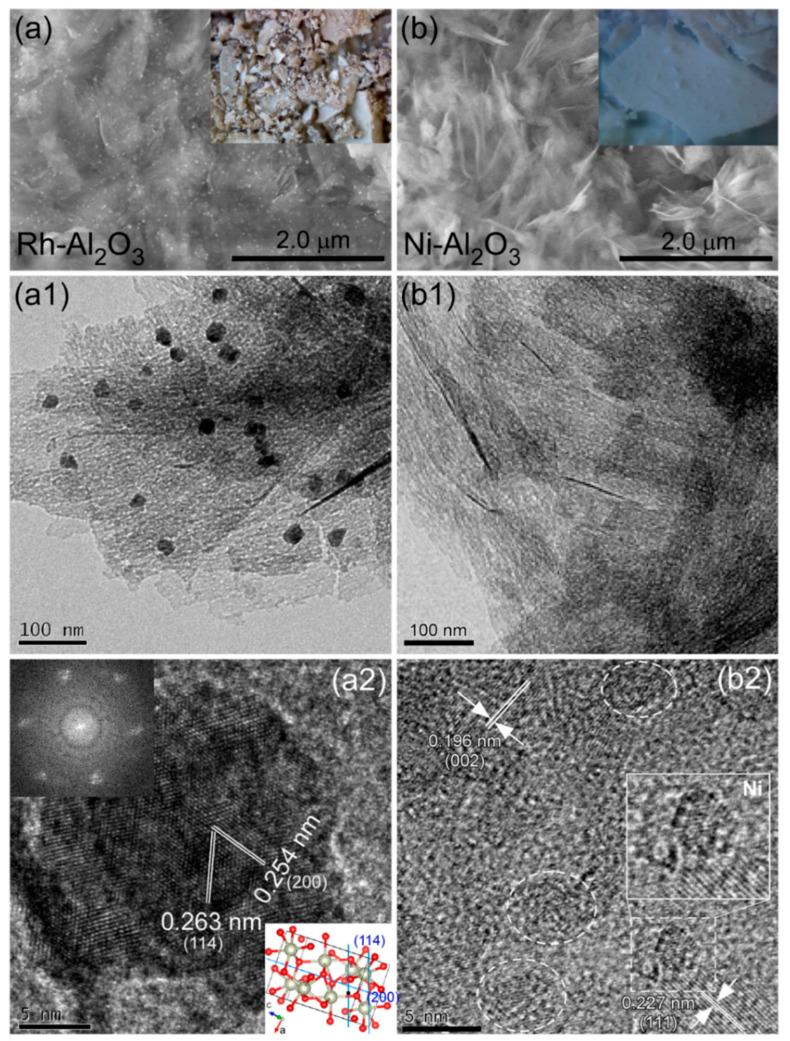
SEM (**a**,**b**), TEM (**a1**,**b1**), HRTEM (**a2**,**b2**) images of selected Rh-Al_2_O_3_ (**a**,**a1**,**a2**) and Ni-Al_2_O_3_ (**b**,**b1**,**b2**) nanosheets. Insets of Figure 2(a2) show the fast Fourier transform (FFT) pattern of the HRTEM image, and the structure projection of the (114) and (200) planes for Rh_2_O_3_.

Figure 2 shows the SEM and TEM images of selected Ni- and Rh-loaded Al_2_O_3_ nanosheets. The SEM images of other M-loaded Al_2_O_3_ nanosheets are provided in the Supporting Information, Figure S1. The SEM image (Figure 2a) of Rh-Al_2_O_3_ showed small nanoparticles embedded on the nanosheets. The nanoparticles appeared as a result of Rh particle formation. The color of burlywood was clearly different from the white color for bare Al_2_O_3_. On the other hand, the SEM image (Figure 2b) of Ni-Al_2_O_3_ nanosheets showed only cotton-like nanosheets. It was difficult to discriminate Ni species from the bare Al_2_O_3_ support. However, the color clearly changed from white to pale blue upon Ni-loading. The photos and optical microscope images of the M-loaded Al_2_O_3_ nanosheets are provided in the Supporting Information, Figures S2 and S3, respectively. Although the SEM images showed no clear metal embedment, the color change was a clear indication of metal-loading on the Al_2_O_3_ support. The metal-loading was also confirmed by the X-ray photoelectron spectroscopy (XPS) data, discussed below.

The TEM images (Figure 2(a1)) of Rh-Al_2_O_3_ clearly showed NPs (with a size of ~20 nm) embedded onto the nanosheets. For the HRTEM image (Figure 2(b2)) of an Rh-NP, clear lattice fringes were observed, and the distances were estimated to be 0.263 nm and 0.254 nm. These distances matched well with the (114) and (200) crystal planes of orthorhombic (Pbca) Rh_2_O_3_ (ICSD ref. 98-000-9206), respectively. This indicated that Rh was embedded not in the metallic form but rather in the oxide form. This was further confirmed by the XPS data below. The fast Fourier transform (FFT) pattern of the HRTEM image reflected the crystallinity of the Rh oxide. The TEM images (Figure 2(b1)) of Ni-Al_2_O_3_ nanosheets showed only nanosheet morphology, consistent with the corresponding SEM image (Figure 2b).

For the HRTEM image in Figure 2(b2), the lattice fringes with distances of 0.227 nm and 0.196 nm matched well with the (111) and (002) crystal planes of the cubic phase γ-Al_2_O_3_. The lattices showed poor crystallinity compared with those of bare Al_2_O_3_, seen in Figure 1(b3). Interestingly, some areas (dotted circles) showed very poor crystallinity, and these appeared like amorphous particles. This is likely an indication of Ni embedment on the Al_2_O_3_ nanosheets. Very similarly for Co-Al_2_O_3_ nanosheets, although particles were not clearly seen in the TEM image (Supporting Information, Figure S4), the corresponding HRTEM image showed the areas with very poor crystallinity. The areas appeared like Co-embedment in the Al_2_O_3_ support.

The BET surface areas of Ni-Al_2_O_3_ and Rh-Al_2_O_3_ nanosheets were measured to be 151.2 m^2^/g and 153.8 m^2^/g, respectively. The surface areas were very similar to that of bare Al_2_O_3_. This indicated that the surface area was not significantly impacted by the metal-loading.

**Figure 3:**
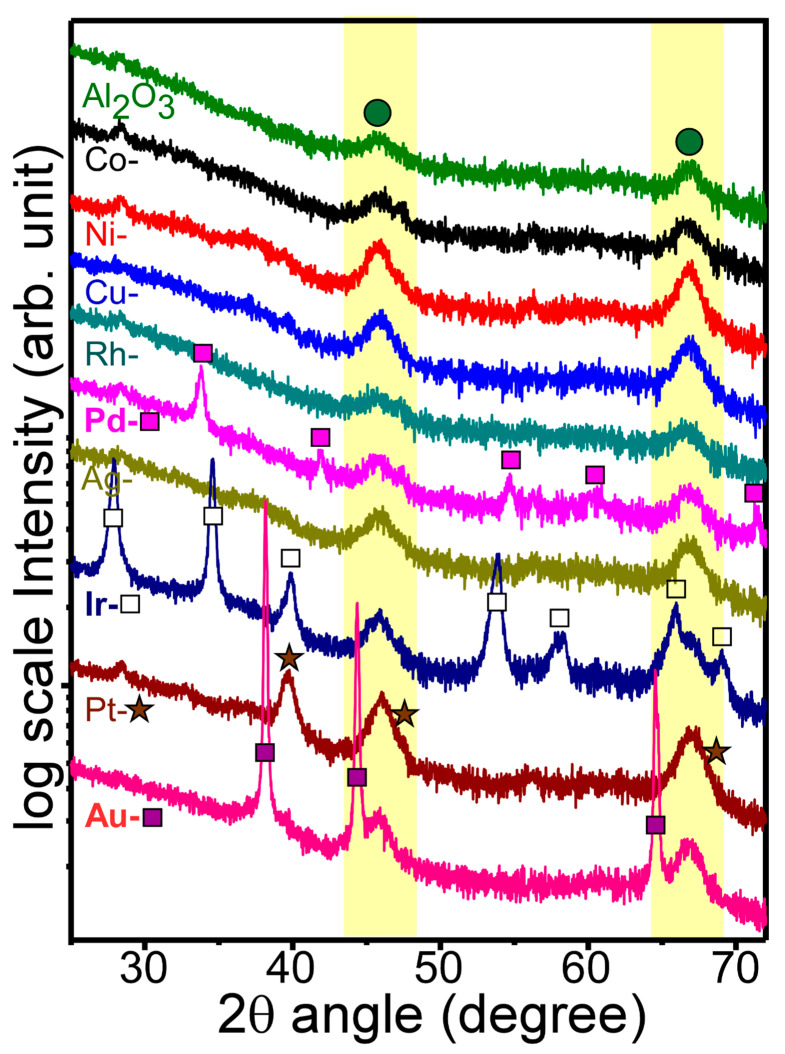
XRD profiles bare Al_2_O_3_ and M-loaded Al_2_O_3_ nanosheets.

Figure 3 displays the X-ray diffraction patterns of bare Al_2_O_3_ and M-loaded Al_2_O_3_ nanosheets. For the XRD patterns of bare Al_2_O_3_, two distinctive peaks were observed at 2θ = 45.9° and 67.0°. These two peaks could be assigned to the (002) and (022) planes of cubic phase (Fm-3m) γ-Al_2_O_3_, (ICSD ref. 98-003-0267), respectively. The XRD result was in good consistency with the HRTEM result of the bare Al_2_O_3_ nanosheet. For the XRD profiles of M-loaded Al_2_O_3_ nanosheets, two peaks were commonly observed, as expected. Interestingly, Co, Ni, Cu, Rh, and Ag-loaded samples showed no significant extra peaks in the corresponding XRD profiles. These results indicated that the metals were loaded with an amorphous oxide state (discussed below in XPS) or embedded very uniformly without forming good crystal phases. In addition, because the metal amount was only 2 mol%, the XRD patterns could not be clearly observed when the phase was an amorphous oxide form. As seen in the HRTEM images of Ni-Al_2_O_3_ and Co-Al_2_O_3_ nanosheets discussed above (Figure 2(b2) and Figure S4, respectively), the particle-like areas showed very poor crystallinity. On the other hand, Pd, Ir, Pt, and Au-loaded samples showed new peaks in the corresponding XRD profiles.

For the XRD patterns of Pd-Al_2_O_3_ nanosheets, several peaks at 2θ = 33.8°, 42.0°, 54.7°, 60.1°, and 71.5° showed good matches with the (011), (110), (112), (013), and (121) crystal planes of tetragonal (p 42/mmc) PdO (ICSD ref. 98-002-9281), respectively [13]. For Ir-Al_2_O_3_ nanosheets, several strong XRD peaks were observed at 2θ = 27.9°, 34.6°, 39.9°, 53.9°, 57.9°, 58.3°, 66.0°, 69.0°, and 73.0°, with good matches with the (110), (011), (020), (121), (220), (002), (130), (112), and (031) crystal planes of tetragonal (p 42/mnm) IrO_2_ (ICSD ref. 98-008-4577), respectively. For Pt-Al_2_O_3_ nanosheets, three major peaks were observed at 2θ = 39.8°, 46.2°, and 67.5°, assigned to the (111), (002,) and (022) crystal planes of the cubic (Fm-3m) crystal phase of metallic Pt (ICSD ref. 98-007-6153), respectively. For Au-Al_2_O_3_ nanosheets, three strong peaks were observed at 2θ = 38.1°, 44.3°, and 64.5°, assigned to the (111), (002), and (022) crystal planes of the cubic (Fm-3m) crystal phase of Au (ICSD ref. 98-061-1624), respectively.

**Figure 4:**
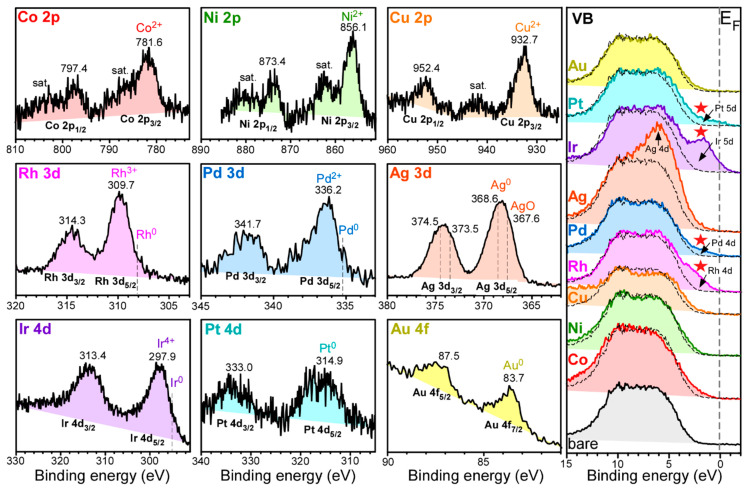
Co 2p, Ni 2p, Cu 2p, Rh 3d, Pd 3d, Ag 3d, Ir 4d, Pt 4d, Au 4d, and VB profiles of Co-Al_2_O_3_, Ni-Al_2_O_3_, Cu-Al_2_O_3_, Rh-Al_2_O_3_, Pd-Al_2_O_3_, Ag-Al_2_O_3_, Ir-Al_2_O_3_, Pt-Al_2_O_3_, and Au-Al_2_O_3_ nanosheets.

XPS was employed to confirm the loading of the transition metals and examine the oxidation states. Figure 4 shows Co 2p, Ni 2p, Cu 2p, Rh 3d, Pd 3d, Ag 3d, Ir 4d, Pt 4d, and Au 4d of Co-Al_2_O_3_, Ni-Al_2_O_3_, Cu-Al_2_O_3_, Rh-Al_2_O_3_, Pd-Al_2_O_3_, Ag-Al_2_O_3_, Ir-Al_2_O_3_, Pt-Al_2_O_3_, and Au-Al_2_O_3_ nanosheets, respectively. XPS valence band spectra (Figure 4, right panel) are also displayed for the corresponding samples. The survey, Al 2p, O 1s, and C 1s profiles are provided in the Supporting Information, Figure S5. All the binding energies (BEs) were referenced to the C 1s XPS peak at 284.8 eV. The survey spectra commonly showed the elements of Al, O, and C (surface impurities), as expected. The XPS peaks of the loaded transition metals were very weakly observed.

For Co-Al_2_O_3_ nanosheets, Co 2p_1/2_ and Co 2p_3/2_ XPS peaks were observed at binding energies (BEs) of 797.4 eV and 781.6 eV, respectively, with a spin-orbit splitting of 15.8 eV. This could be attributed to Co^2+^ of CoO and Co(OH)_2_ [31,32]. The corresponding satellite peaks for Co^2+^ were clearly observed around 803 eV and 786 eV. For Ni-Al_2_O_3_ nanosheets, Ni 2p_1/2_ and Ni 2p_3/2_ XPS peaks were observed at binding energies (BEs) of 873.4 eV and 856.1 eV, respectively, with a spin-orbit splitting of 17.3 eV. This could be attributed to Ni^2+^ of NiO and Ni(OH)_2_ [15,31,32]. The corresponding satellite peaks for Ni^2+^ were clearly observed around 880 eV and 862 eV. For Cu-Al_2_O_3_ nanosheets, Cu 2p_1/2_ and Cu 2p_3/2_ XPS peaks were observed at binding energies (BEs) of 952.4 eV and 932.7 eV, respectively, with a spin-orbit splitting of 19.7 eV. This could be attributed to Cu^2+^ of CuO and Cu(OH)_2_ [32,33]. The corresponding satellite peak for Cu^2+^ was clearly observed around 942 eV. For Co, Ni, and Cu, no metallic XPS peaks were observed. On the basis of XRD, HRTEM, and XPS data, Co, Ni, and Cu appeared to be embedded as an amorphous oxide form.

For Rh-Al_2_O_3_ nanosheets, Rh 3d_3/2_ and Rh 3d_5/2_ XPS peaks were observed at BEs of 314.3 eV and 309.7 eV, respectively, with a spin-orbit splitting of 4.6 eV. The XPS BEs were attributed to an oxidation state of Rh^3+^ [32,34]. As discussed above, the lattice distances in the HRTEM image confirmed orthorhombic Rh_2_O_3_. An additional weak shoulder peak was seen around 308 eV for Rh 3d_5/2_ peak. This could be due to metallic Rh [32,34]. On the basis of the XPS and HRTEM data, Rh-species appeared to be consistent with Rh@Rh_2_O_3_ core-shell type structure.

For Pd-Al_2_O_3_ nanosheets, Pd 3d_3/2_ and Pd 3d_5/2_ XPS peaks were observed at BEs of 341.7 eV and 336.2 eV, respectively, with a spin-orbit splitting of 5.5 eV. The XPS peaks were attributed to an oxidation state of Pd^2+^ [32,34,35]. There was a good coincidence between the oxidation state of the XPS and the XRD profiles of tetragonal PdO. A weak shoulder of the Pd 3d_5/2_ peak was seen around 335.5 eV, plausibly due to metallic Pd [32]. For Ag-Al_2_O_3_ nanosheets, Ag 3d_3/2_ and Ag 3d_5/2_ XPS peaks were observed at BEs of 374.5 eV and 368.6 eV, respectively. This was attributed to metallic Ag [6,32,36]. The shoulder XPS peak at 367.6 eV for Ag 3d_5/2_ was plausibly due to AgO [36]. On the basis of the XPS profile for each M-Al_2_O_3_ sample, it could be concluded that the transition metal was loaded on the Al_2_O_3_ support.

For Ir-Al_2_O_3_ nanosheets, Ir 4d_3/2_ and Ir 4d_5/2_ XPS peaks were observed at BEs of 313.6 eV and 297.7 eV, respectively. These peaks were assigned to the Ir^4+^ oxidation state [37], in good coincidence with the XRD profiles of tetragonal IrO_2_, shown above. A weak shoulder of Ir 4d_5/2_ peak was seen around 295 eV, plausibly due to metallic Ir [37]. For Pt-Al_2_O_3_ nanosheets, Pt 4d_3/2_ and Pt 4d_5/2_ XPS peaks were observed at BEs of 332.9 eV and 314.9 eV, respectively, with a spin-orbit splitting of 18.0 eV. The XPS peaks were attributed to metallic Pt [34,35], which was well-fitting with the XRD result of metallic Pt. For Au-Al_2_O_3_ nanosheets, the Au 4f_7/2_ and Au 4f_5/2_ XPS peaks were observed at BEs of 87.3 eV and 83.6 eV, respectively, with a spin-orbit splitting of 3.7 eV. The XPS peaks were attributed metallic Au [32]. This result was in good agreement with the XRD profiles of metallic Au, shown above.

For the Al 2p XPS profiles (Supporting Information, Figure S5), a broad peak was commonly observed around 74.1 eV, attributed to Al of the Al_2_O_3_ support [32,38]. An additional peak at 75.0 eV was observed and attributed to the Al of surface Al-OH species [32,38]. For the O 1s XPS profiles (Supporting Information, Figure S5), a broad peak was commonly observed around 530.9 eV due to lattice O of Al_2_O_3_ support. A broad shoulder at 532.5 eV was attributed to oxygen defects and surface OH/H_2_O species [39].

The valence band (VB) spectra are shown in Figure 4 to further examine electronic structures. For the VB of bare Al_2_O_3_ nanosheets, two broad features were seen around 9 eV and 6 eV, attributed bonding 2pσ (mixed with Al 3s, Al 3p, and Al 3d) and antibonding 2pπ of the oxygen [40]. For VB spectra of M-Al_2_O_3_ nanosheets, the density of states (DOS) was observed to be closer to the Fermi level. Especially, Rh, Pd, Ir, and Pt showed more clearly new features near 2 eV below the Fermi level, attributed to the Rh 4d, Pd 4d, Ir 5d, and Pt 5d, respectively. This could be related to the higher CO oxidation activities for these metals, discussed below. However, the DOS profiles showed no explicit relationship with the photocatalytic CO_2_ reduction activity. The detailed roles of the overlayer elements could be understood with the aid of density functional theory.

**Figure 5:**
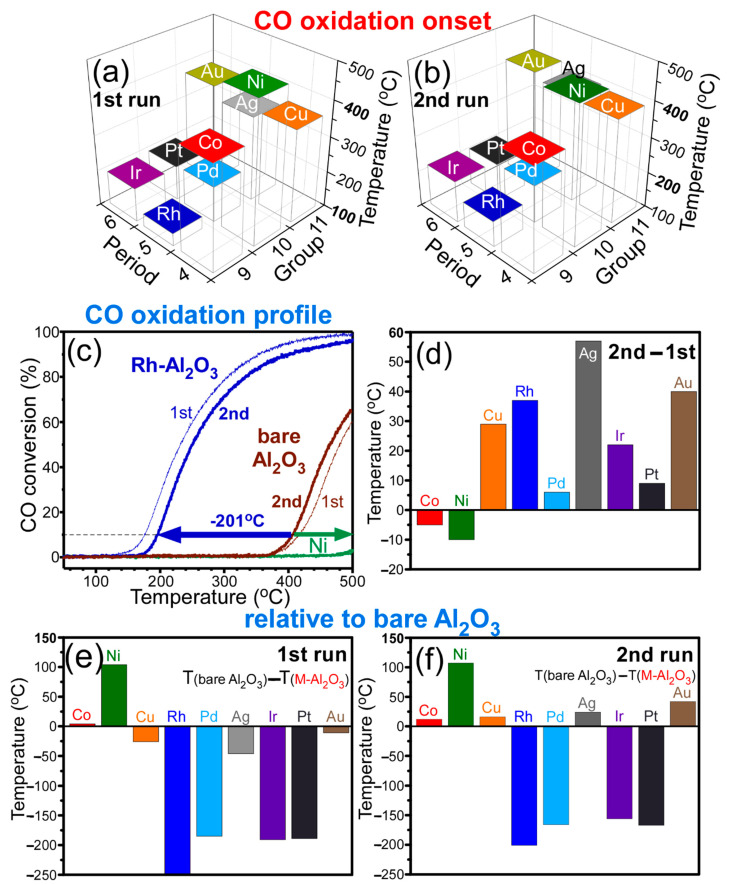
CO oxidation onsets for the first (**a**) and second (**b**) runs of M-loaded Al_2_O_3_ nanosheets. Selected first and second run CO oxidation profiles with temperature (**c**) for bare, Rh- and Ni-Al_2_O_3_ nanosheets. Differences in CO oxidation onsets for the first and the second runs (**d**) and relative CO oxidation onset temperatures relative to that of bare nanosheets (**e**,**f**).

Temperature-programmed CO oxidation profiles (Supporting Information, Figure S6) were obtained to examine thermal CO oxidation catalytic activities for bare Al_2_O_3_ and M-Al_2_O_3_ nanosheets. To evaluate the catalytic activities of the catalysts, Figure 5a,b display the CO oxidation onset temperatures for the first and the second runs, respectively. Table 1 summarizes the onset temperatures (T_M-Al2O3__,onset_) and the temperature difference (T_M-Al2O3,2nd_ − T_M-Al2O3,1st_) between the first and the second runs. The onset temperatures of Ir-, Pt-, Pd-, and Rh-loaded Al_2_O_3_ nanosheets were observed to be much lower than those of Au-, Ag-, Cu-, Co-, and Ni-loped Al_2_O_3_ nanosheets. The group 11 (Au, Ag, and Cu) and the period 4 (Co, Ni, and Cu) elements showed much poor catalytic activity on the Al_2_O_3_ support. Additionally, the onset temperatures of Au and Ag-loaded Al_2_O_3_ nanosheets were unexpectedly even higher than expected [6,10,15]. In other words, the Au- and Ag-loaded Al_2_O_3_ nanosheets showed poorer CO oxidation activity. In the first run, the Rh-Al_2_O_3_ nanosheets showed the lowest onset of 135 °C, while the Ni-Al_2_O_3_ nanosheets showed the highest onset of 490 °C. The temperature difference between the two samples was estimated to be 335 °C. In the second run, the Rh-Al_2_O_3_ nanosheets also showed the lowest onset of 172 °C while the Ni-Al_2_O_3_ nanosheets showed the highest onset of 480 °C. The temperature difference was estimated to be 308 °C. Pd, Ir, and Pt showed the CO oxidation onsets at 207 °C, 217 °C, and 216 °C, respectively, in the second run. For highly dispersed (or single atom state) 0.2 wt % Pt on mesoporous Al_2_O_3_ support, Zhang et al. reported CO oxidation onset at ~200 °C, which was in good coincidence with the present result [5]. These results clearly indicated that the CO oxidation activity was highly influenced by the nature of overlayer metal species.

Figure 5c shows the CO oxidation profiles for the first and the second runs of the selected samples (bare Al_2_O_3_, Ni-Al_2_O_3_, and Rh-Al_2_O_3_ catalysts). As seen in the Figure 5, the CO oxidation onset of Rh-Al_2_O_3_ occurred much earlier than that of bare Al_2_O_3_. The onsets of Rh-Al_2_O_3_ in the first and the second runs were observed to be 251 °C and 201 °C lower than those of bare Al_2_O_3_, respectively. However, the onset temperatures became much higher upon loading Ni.

To examine the difference in catalytic activity between the first and the second runs, Figure 5d plots the temperature differences (T_M-Al2O3__,2nd_ − T_M-Al2O3__,1st_*)* in the CO oxidation onsets between the first and the second runs. In the first run, the CO oxidation reactions were performed with the as-prepared samples. In the second run, the CO oxidation reactions were performed with samples, which were already participated in the first run. Therefore, the surface states (or the catalytic-active sites) were expected to be different for the samples in the first and the second runs. The values (T_M-Al2O3__,2nd_ − T_M-Al2O3__,1st_) are summarized in Table 1. The positive value (Figure 5d) indicated that the CO oxidation started at a higher temperature in the second run. In other words, the CO oxidation catalytic activity became lower in the second run.

For Co- and Ni-Al_2_O_3_ nanosheets, the onset temperatures in the second run were observed to be slightly lower than those in the first run. However, the other samples commonly showed higher onset temperatures in the second run, compared with the first run. This indicated that, for the latter, the catalytic activity became somewhat lower after the first run. The lower catalytic activity appeared to be mainly due to a change in crystallinity and lower catalytic-active sites.

To evaluate the roles of the transition metals in catalytic activities, compared with bare Al_2_O_3_, Figure 5e,f show the relative CO oxidation onsets (T_Al_2___O_3__ − T_M-Al_2___O_3__), compared with those of the first and the second runs of the bare Al_2_O_3_, respectively. The values are summarized in Table 2. In the first runs, the T_Al2O3,1st_ − T_M-Al2O3,1st_ values of Co and Ni showed positive, and others showed negative values. In the second runs, Co, Ni, Cu, Ag, and Au showed positive, and others showed negative values. On the basis of Figure 5e,f, the catalytic activity became poorer upon loading Co and Ni, compared with bare Al_2_O_3_. Unexpectedly, the Au, Ag, and Cu (group 11) showed somewhat higher activities in the first run but showed poorer catalytic activity in the second run, compared with the bare Al_2_O_3_ nanosheet. The Rh, Pd, Ir, and Pt showed much higher (with lowering of onset temperatures between 156 °C and 261 °C) CO oxidation activity in the first and second runs. Conclusively, the CO oxidation activity showed the order of Ni < Co < Au < Cu < Ag < Pd < Pt < Ir < Rh in the first run, and Ni < Au < Ag < Cu < Co < Ir < Pt ≈ Pd < Rh in the second run.

For CO oxidation, a simplified mechanism is described below;

M-Al_2_O_3_ + CO (g) → CO(ad)-M-Al_2_O_3_      adsorption of CO (1)

M-Al_2_O_3_ + 1/2O_2_ (g) → O(ad)-M-Al_2_O_3_   dissociative adsorption of O_2_ (2)

CO (g) + O(ad)-M-Al_2_O_3_ → M-Al_2_O_3_ + CO_2_ (g) (3)

CO (g) + HO-M-Al_2_O_3_ → M-Al_2_O_3_-H + CO_2_ (g) (4)

CO (g) + O_2_(ad)-M-Al_2_O_3_ → CO_3_(ad)-M-Al_2_O_3_   carbonate formation (5)

CO_3_(ad)-M-Al_2_O_3_ → O(ad)-M-Al_2_O_3_ + CO_2_ (g) (6)

The CO oxidation mechanism was explained by the Langmuir-Hinshelwood mechanism [12,13] and the non-Langmuir-Hinshelwood mechanism [4,11], depending on the overlayer transition metals. In reaction (1), CO was adsorbed on metal site, and in reaction (2), oxygen was dissociatively adsorbed on the surface. In reaction (3), gaseous CO and surface O reacted to release CO_2_ [12,13]. When moisture was present in the reaction, the surface OH group was plausibly formed and CO might also react with the surface metal hydroxide to form the CO_2_ in reaction (4) [15]. On the basis of the FT-IR spectra (Supporting Information, Figure S7), surface OH groups were observed in the as-prepared samples. Therefore, reaction (4) was likely involved in the first run of CO_2_ formation. If H was not desorbed as H_2_O, the surface H was recycled as shown in reaction (4). Otherwise, the H was removed from the surface as gaseous H_2_O, and, thus, the reaction (4) was diminished in the second run. Reaction (5) was also reported for surfaces such as Pt/Al_2_O_3_ [4,11]. In reaction (5), CO was adsorbed on oxygenated metal atoms to initially form carbonate. Then, the carbonate dissociated to generate CO_2_ in reaction (6).

**Figure 6:**
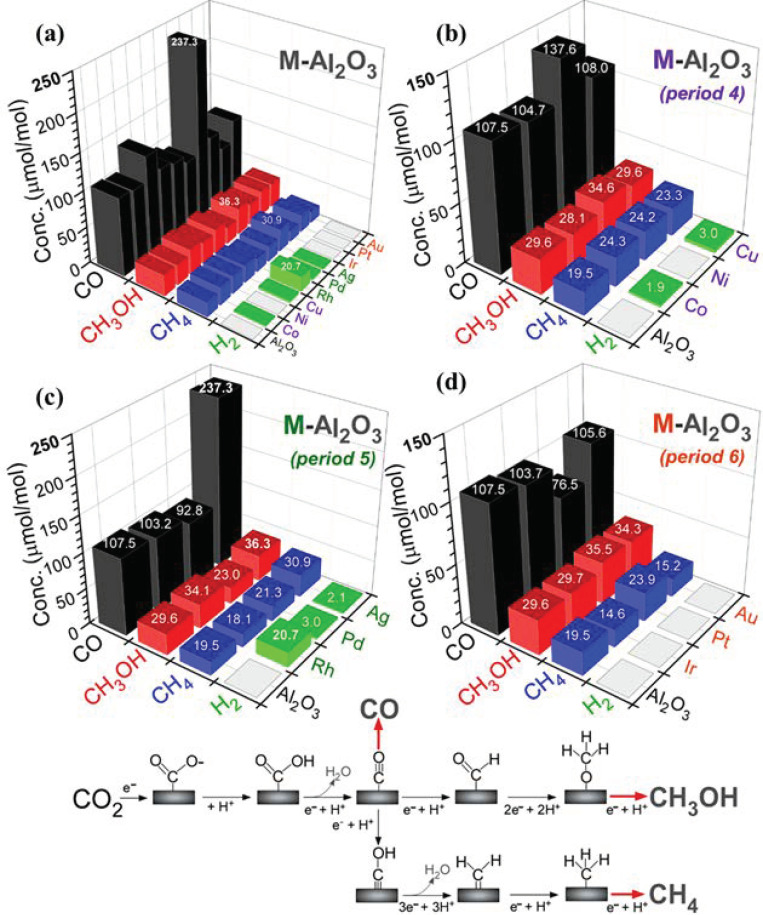
CO_2_ reduction CO, CH_4_, and CH_3_OH yields (μmol/mol) over bare and M-loaded Al_2_O_3_ nanosheets (**a**), (Co, Ni, and Cu)-Al_2_O_3_ (**b**), (Rh, Pd, and Ag)-Al_2_O_3_ (**c**), (Ir, Pt, and Au)-Al_2_O_3_ (**d**), and CO_2_ reduction mechanism.

Photocatalytic CO_2_ reduction products were examined for bare Al_2_O_3_ and M-Al_2_O_3_ nanosheets and are displayed in Figure 6 [39,41,42]. Major CO_2_ reduction products were observed to be carbon monoxide (CO), methanol (CH_3_OH), and methane (CH_4_) with an order: CH_4_ < CH_3_OH < CO. CO was the most dominantly produced species. CH_3_OH showed a higher production amount compared with CH_4_. Hydrogen (H_2_) was additionally observed as a photocatalytic water splitting product during CO_2_ reduction. Figure 6a plots all of the product amounts (μmol/mol = ppm) for bare Al_2_O_3_ and M-Al_2_O_3_ nanosheets. As a quick glance, Ag-Al_2_O_3_ nanosheets showed the highest amounts of CO_2_ reduction products: 237.3 ppm for CO, 36.3 ppm for CH_3_OH, and 30.9 ppm for CH_4_, and Rh-Al_2_O_3_ nanosheets showed the highest H_2_ production (20.7 ppm). For the bare Al_2_O_3_ nanosheets in Figure 6b, CO, CH_3_OH, and CH_4_ were observed to be 107.5 ppm, 29.6 ppm, and 19.5 ppm, respectively. No H_2_ was detected. CO reduction yields (μmol/mol) in different groups of 9, 10, and 11, and with different units (μmol/g), are provided in the Supporting Information, Figures S8 and S9, respectively.

For bare Al_2_O_3_, the selectivities for CO, CH_3_OH, and CH_4_ were estimated to be 68.6%, 18.9%, and 12.5%, respectively. Upon Co- and Cu-loading, CH_4_ and H_2_ showed meaningful (>25%) enhancements. However, the amounts of CO and CH_3_OH showed no critical change. CO, CH_3_OH, and CH_4_ productions were enhanced by 28%, 17%, and 24% upon Ni-loading. CO was increased by 2.2× upon loading Ag in Figure 6c. CH_3_OH and CH_4_ were also increased by 1.23× and 1.58×, respectively, upon loading Ag. Rh and Pd-loadings had a smaller effect on the CO production relative to the bare support. CH_3_OH and CH_4_ productions were not meaningfully enhanced by Rh- and Pd-loadings. Instead, interestingly the H_2_ production was commonly observed in these metal-loadings. For Ir, Pt, and Au elements in period 6, CO productions were all decreased by metal-loadings. CH_3_OH productions were somewhat increased by 19% and 16% upon loading of Pt and Au, respectively. The CH_4_ production was only increased upon loading Pt relative to the bare substrate.

For H_2_ production, Ag, Pd, and Rh (in period 5) metals commonly showed H_2_ productions with amounts of 2.1 ppm, 3.0 ppm, and 20.7 ppm, respectively. For the metals of Co, Ni, and Cu (in period 4), the H_2_ production amounts were observed to be 1.9 ppm, 0 ppm, and 3.0 ppm, respectively. That is, Ni showed no H_2_ production. The metals of Au, Pt, and Ir in period 6 commonly showed no H_2_ production at all. The Rh-Al_2_O_3_ nanosheets predominantly showed the highest H_2_ production with an amount of 20.7 ppm.

The photocatalytic CO_2_ reduction mechanism is generally written as *x*CO_2_ + *y*H^+^ + *z*e^−^ → C*_a_*H*_b_*O*_c_* products + *d*H_2_O [41,42]. Electrons (e^−^) and holes (h^+^) were generated under UVC irradiation in reaction (7). H^+^ ion was generated via the reactions in (8)–(11). The generation of electrons was an important factor for the multielectron processes. The mechanisms for the productions of CO (in reaction (12)), CH_3_OH (in reaction (13)), and CH_4_ (in reaction (14)) are written as below and shown in Figure 6 [38,39].

Al oxides + UVC → Al oxides (e^−^ + h^+^) (7)

H_2_O → H^+^ + OH (8)

OH^−^ + h^+^ → •OH (9)

•OH + H_2_O + 3h^+^ → O_2_ + 3H^+^ (10)

H^+^ + e^−^ → 1/2H_2_ (11)

CO_2_ + 2H^+^ + 2e^−^ → CO + H_2_O, −0.530 V vs. standard hydrogen electrode (SHE) (12)

CO_2_ + 6H^+^ + 6e^−^ → CH_3_OH + H_2_O, −0.380 V vs. SHE (13)

CO_2_ + 8H^+^ + 8e^−^ → CH_4_ + 2H_2_O, −0.240 V vs. SHE (14)

These reaction channels were closely spaced in free energy change, and, thus, the hydrogen production channel (H^+^ + e^−^ → 1/2H_2_, −0.42 V vs. SHE) occurred competitively. In the mechanism, CO_2_ was initially adsorbed to form COOH. The COOH was then attacked by H^+^ and e^−^ to generate gaseous CO. The CO production channel was only enhanced by loading Ag or Ni on Al_2_O_3_ support. CH_3_OH production was likely formed when surface CO_ad_ underwent step-wise hydrogenation. This production was enhanced by loading Ni, Rh, Ag, Pt, or Au on Al_2_O_3_ support. CH_4_ production was formed via C–O bond scission of hydrogenated ≡C‒OH and new C‒H bond formation. This production was somewhat enhanced by loading Co, Ni, Cu, Ag, or Pt. The present pre-screening tests need further investigations to understand the detailed roles of the overlayer elements, with the aid of density functional theory.

## 4. Conclusions

In summary, γ-Al_2_O_3_ nanosheets were prepared by the solvothermal method followed by thermal calcination at 600 °C for 2 h. Transition metals (M = Co, Ni, Cu, Rh, Pd, Ag, Ir, Pt, and Au) were loaded on Al_2_O_3_ nanosheet supports, and their thermal CO oxidation and photocatalytic CO_2_ reduction activities were fully tested.

The thermal CO oxidation activity showed the order of Ni < Co < Au < Cu < Ag << Pd < Pt < Ir < Rh in the first run, and Ni < Au < Ag < Cu < Co << Ir < Pt ≈ Pd < Rh in the second run. The Au, Ag, Co, Ni, and Cu elements reduced the catalytic activity on the Al_2_O_3_ support. CO oxidation activity was greatly enhanced by the loading of Ir, Pt, Pd, and Rh elements. Rh-Al_2_O_3_ nanosheets showed the highest CO oxidation activity with onset temperatures of 135 °C and 172 °C for the first and the second runs, respectively.

Photocatalytic CO_2_ reduction experiments were also performed to show that CO, CH_3_OH, and CH_4_ were common products with an order of CH_4_ (14.6–30.9 ppm range) < CH_3_OH (23.0–36.3 ppm range) << CO (76.5–237.3 ppm range). The highest performance was achieved after Ag-loadings with yields of 237.3 ppm for CO, 36.3 ppm for CH_3_OH, and 30.9 ppm for CH_4_, corresponding to 2.2×, 1.2×, and 1.6× enhancements, respectively, compared with those for the bare Al_2_O_3_. CO production was substantially decreased by the loading of Pd and Pt. Hydrogen production was enhanced by Rh-loadings with a yield of 20.7 ppm. Conclusively, Rh-Al_2_O_3_ and Ag-Al_2_O_3_ showed the best thermal CO oxidation and photocatalytic CO_2_ reduction performances, respectively, among Co, Ni, Cu, Rh, Pd, Ag, Ir, Pt, and Au element loadings.

The present pre-screening test results could be a useful quick guide for the selection of overlayer transition metals in the groups of 9, 10, and 11 when Al_2_O_3_ is used as a support catalyst material. It also enriched the understanding of the role of an overlayer transition metal.

## Figures and Tables

**Table 1:** CO oxidation onset temperatures (T_M-Al2O3,onset_) in the first and second runs. Differences in CO oxidation onset temperatures (T_M-Al2O3,2nd_ − T_M-Al2O3,1st_) between the first and second runs.

Group #9	First Run	Second Run	Diff.	Group #10	First Run	Second Run	Diff.	Group #11	First Run	Second Run	Diff.
Co	390	385	−5	Ni	490	480	−10	Cu	360	389	29
Rh	135	172	37	Pd	201	207	6	Ag	340	397	57
Ir	195	217	22	Pt	197	206	9	Au	375	415	40

**Table 2:** Differences in CO oxidation onset temperatures (T_Al2O3,onset_ − T_M-Al2O3,onset_) in the first and second runs, compared with that of bare Al_2_O_3_ nanosheets. The CO oxidation onset temperatures of bare Al_2_O_3_ were 386 °C and 373 °C for the first and the second runs, respectively.

Group #9	First Run	Second Run	Group #10	First Run	Second Run	Group #11	First Run	Second Run
Co	4	12	Ni	104	107	Cu	−26	16
Rh	−251	−201	Pd	−185	−166	Ag	−46	24
Ir	−191	−156	Pt	−189	−167	Au	−11	42

## Data Availability

Data are available in the main text.

## Supplementary Materials

The following are available online at https://www.mdpi.com/article/10.3390/nano11051278/s1, Figure S1: Scanning electron microscope (SEM) images for M-loaded Al_2_O_3_ nanosheets, Figure S2: Photos for M-loaded Al_2_O_3_ nanosheets, Figure S3: Optical microscope images for Al_2_O_3_ and M-loaded Al_2_O_3_ nanosheets, Figure S4: Transmission electron microscopic (TEM) and high-resolution TEM (HRTEM) images of Co-Al_2_O_3_ nanosheets, Figure S5: First and second CO oxidation profiles for Al_2_O_3_ and M-loaded Al_2_O_3_ nanosheets, Figure S6: Survey, C 1s, Al 2p, and O 1s profile for bare and M-Al_2_O_3_ nanosheets, Figure S7: FT-IR spectra for Al_2_O_3_ and M-loaded Al_2_O_3_ nanosheets before and after CO oxidation, Figure S8: CO_2_ reduction CO, CH_4_, and CH_3_OH yields (μmol/mol) over bare and M-loaded Al_2_O_3_ nanosheets, group 9: (Co, Rh and Ir)-Al_2_O_3_, group 10: (Ni, Pd and Pt)-Al_2_O_3_, and group 11: (Ir, Pt and Au)-Al_2_O_3_, Figure S9: CO, CH_4_, and CH_3_OH yields (μmol/g) for over bare and M-loaded Al_2_O_3_ nanosheets, (Co, Ni and Cu)-Al_2_O_3_, (Rh, Pd and Ag)-Al_2_O_3_, (Ir, Pt and Au)-Al_2_O_3_.
