# Supplementary material for: Thermal CO Oxidation and Photocatalytic CO2 Reduction over Bare and M-Al2O3 (M = Co, Ni, Cu, Rh, Pd, Ag, Ir, Pt, and Au) Cotton-Like Nanosheets
Source: Nanomaterials (Basel). 2021 May 13;11(5):1278. doi: 10.3390/nano11051278 (PMC8152517; doi:10.3390/nano11051278)
Supplement: Supplementary file 1 [file nanomaterials-11-01278-s001.zip › nanomaterials-1183851-supplementary.pdf]

nanomaterials

MDPI

Supplementary Materials

Thermal CO Oxidation and Photocatalytic CO_2 Reduction over Bare and M-Al_2O_3 (M = Co, Ni, Cu, Rh, Pd, Ag, Ir, Pt, and Au) Cotton-Like Nanosheets

Hee Jung Yoon [1,†], Ju Hyun Yang [2,3,†], So Jeong Park [2] and Youngku Sohn [1,2,3,*]

[1] Department of Chemistry, Yeungnam University, Gyeongsan 38541, Korea; hohoya0102@naver.com
[2] Department of Chemistry, Chungnam National University, Daejeon 34134, Korea; mil03076@naver.com (J.H.Y.); jsjs5921@naver.com (S.J.P.)
[3] Department of Chemical Engineering and Applied Chemistry, Chungnam National University, Daejeon 34134, Korea
* Correspondence: youngkusohn@cnu.ac.kr; Tel.: +82-42-821-6548
† These authors equally contributed to this work.

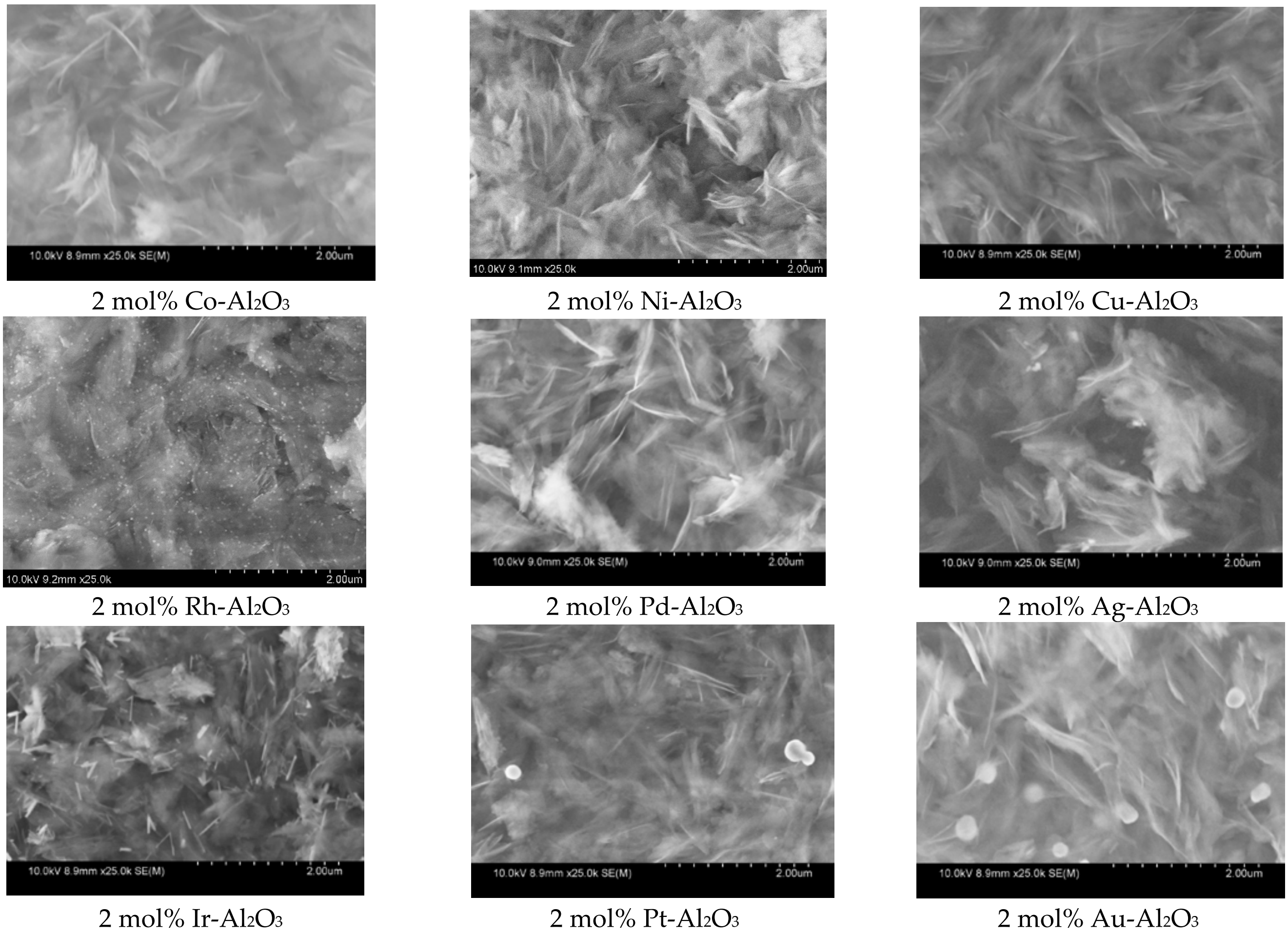

Figure S1. Scanning electron microscope (SEM) images for M-loaded Al_2O_3 nanosheets.

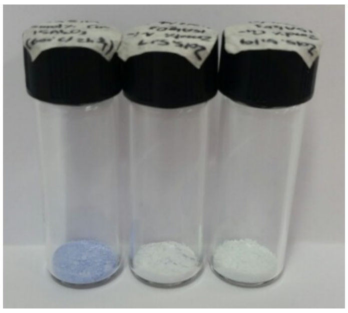

2 mol% Co, Ni, Cu-Al_2O_3

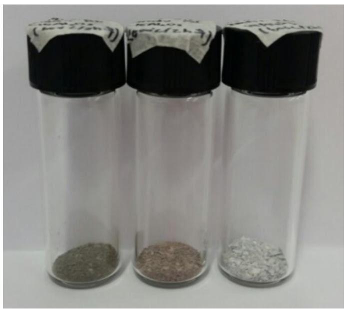

2 mol% Rh, Pd, Ag-Al_2O_3

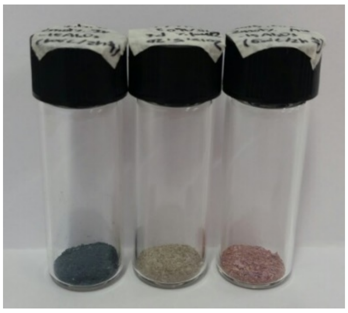

2 mol% Ir, Pt, Au-Al_2O_3

Figure S2. Photos for M-loaded Al2O3 nanosheets.

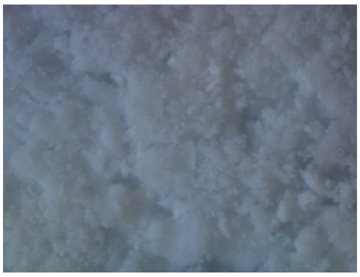

Al_2O_3

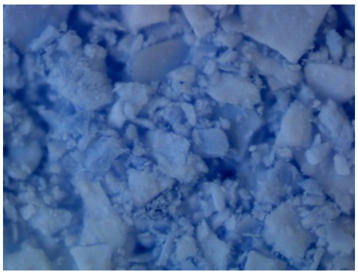

2 mol% Co-Al_2O_3

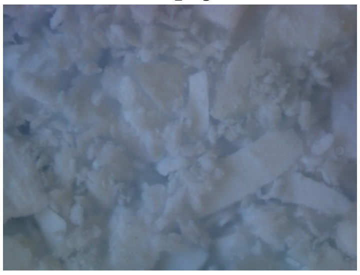

2 mol% Ni-Al_2O_3

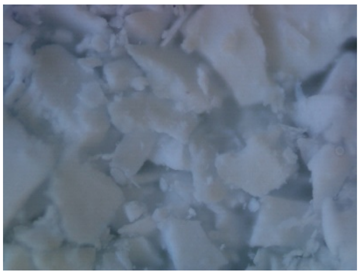

2 mol% Cu-Al_2O_3

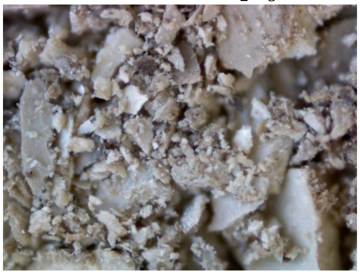

2 mol% Rh-Al_2O_3

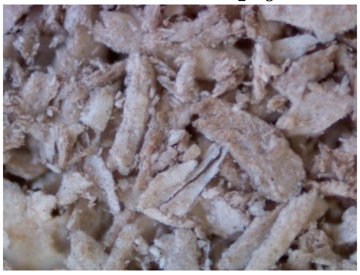

2 mol% Pd-Al_2O_3

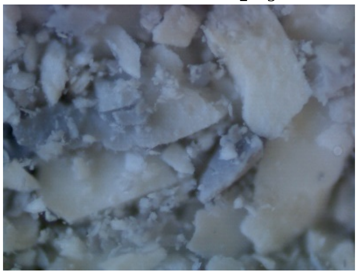

2 mol% Ag-Al_2O_3

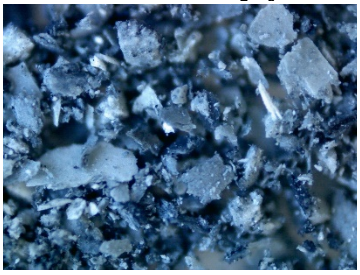

2 mol% Ir-Al_2O_3

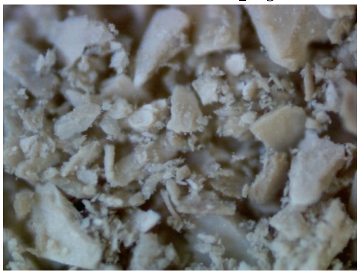

2 mol% Pt-Al_2O_3

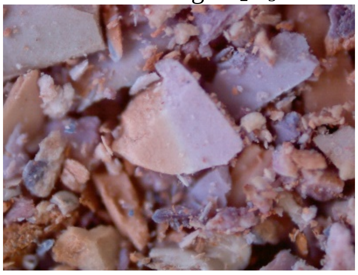

2 mol% Au-Al_2O_3

Figure S3. Optical microscope images for Al_2O_3 and M-loaded Al_2O_3 nanosheets.

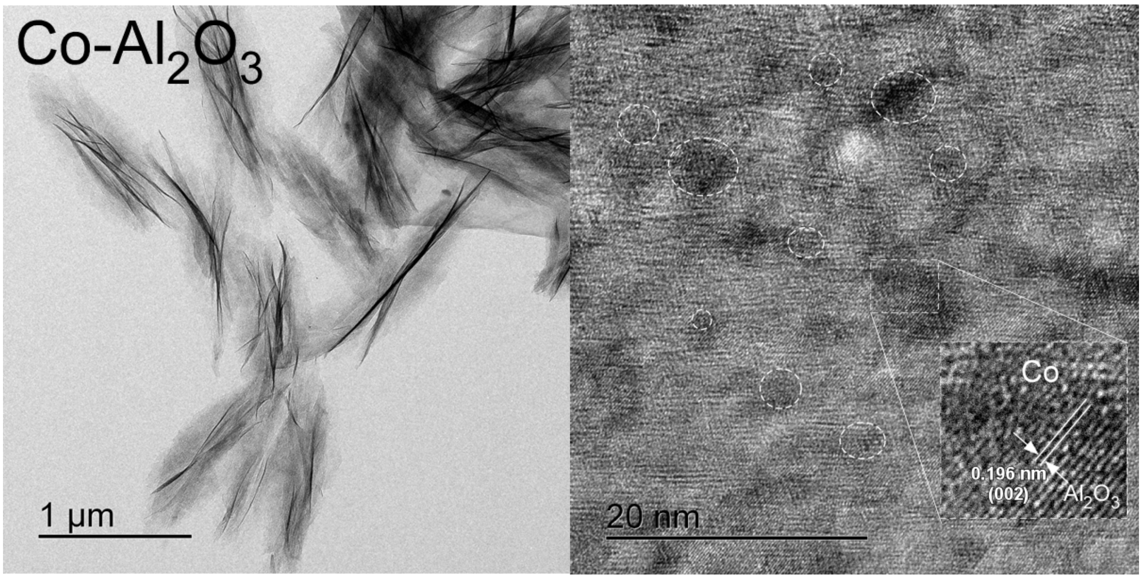

Figure S4. Transmission electron microscopic (TEM) and high-resolution TEM (HRTEM) images of Co-Al_2O_3 nanosheets.

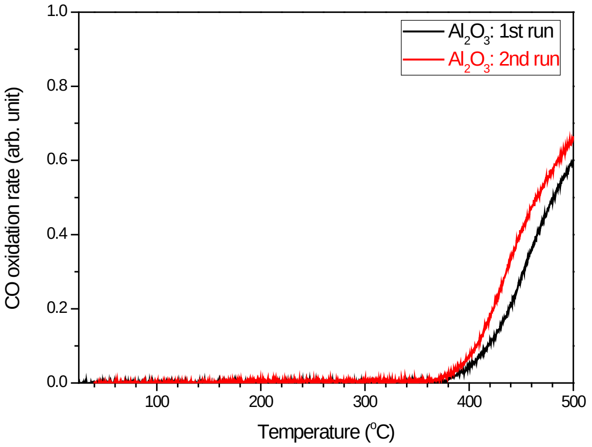

bare Al_2O_3

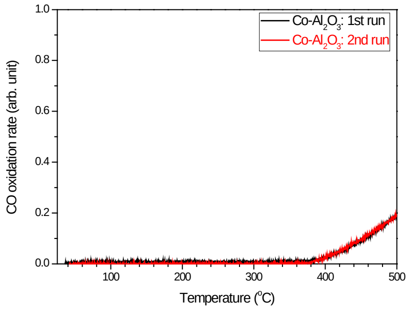

2 mol% Co-Al_2O_3

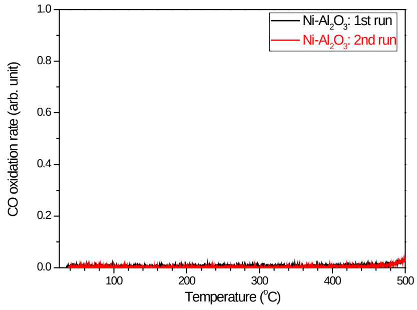

2 mol% Ni-Al_2O_3

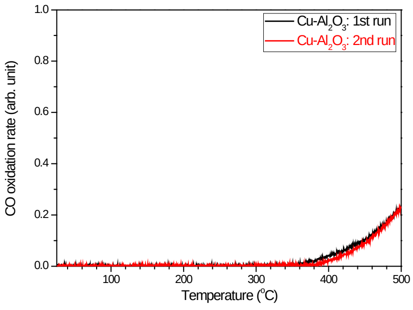

2 mol% Cu-Al_2O_3

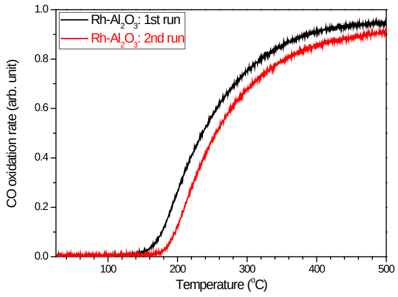

2 mol% Rh-Al_2O_3

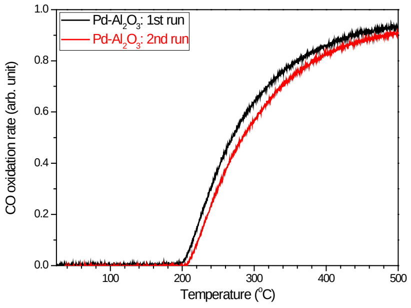

2 mol% Pd-Al_2O_3

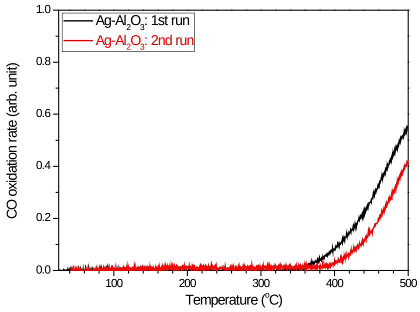

2 mol% Ag-Al_2O_3

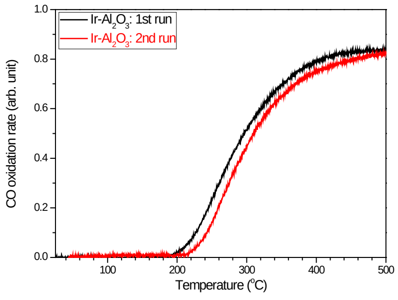

mol% Ir-Al_2O_3

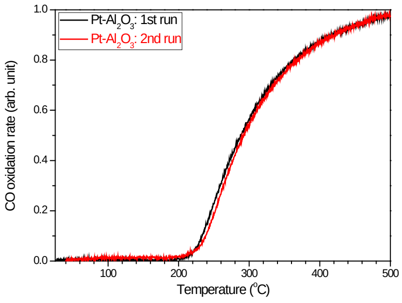

2 mol% Pt-Al_2O_3

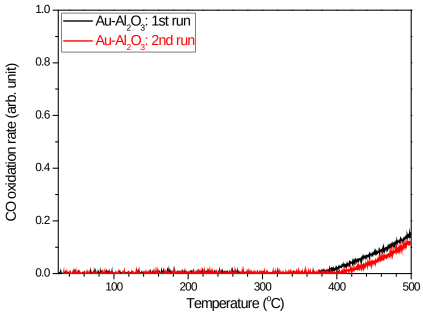

2 mol% Au-Al_2O_3

Figure S5. First and second CO oxidation profiles for Al_2O_3 and M-loaded Al_2O_3 nanosheets.

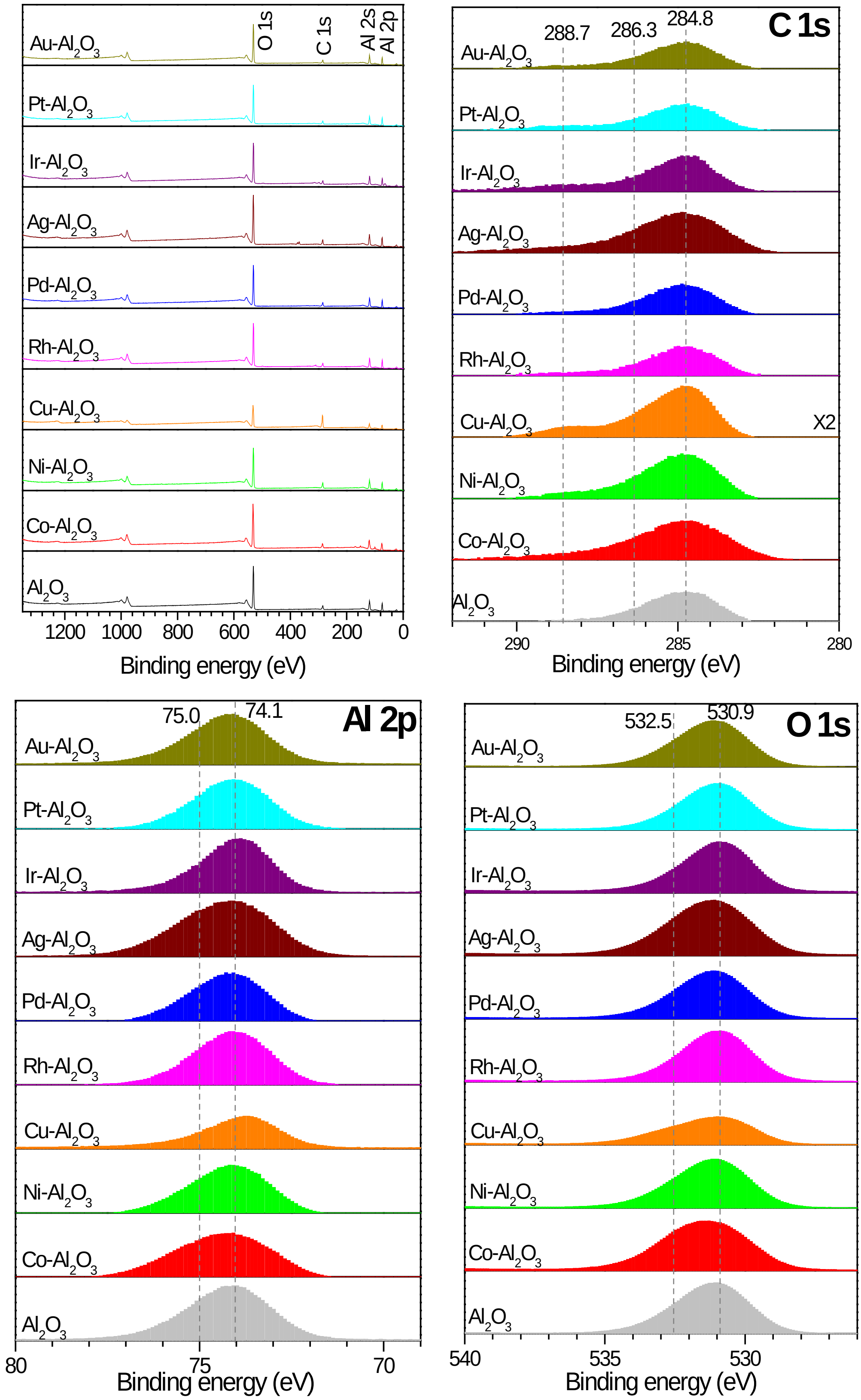

Figure S6. Survey, C 1s, Al 2p, and O 1s profile for bare and M-Al_2O_3 nanosheets.

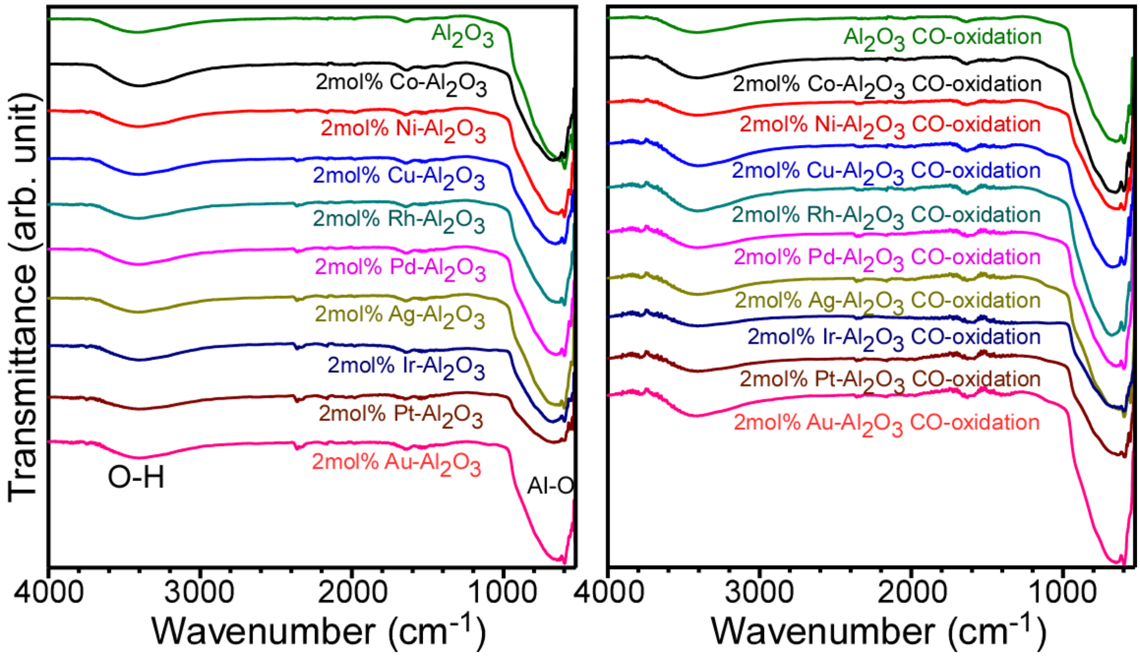

Figure S7. Fourier-transform infrared spectroscopy (FT-IR) spectra for Al_2O_3 and M-loaded Al_2O_3 nanosheets before and after CO oxidation.

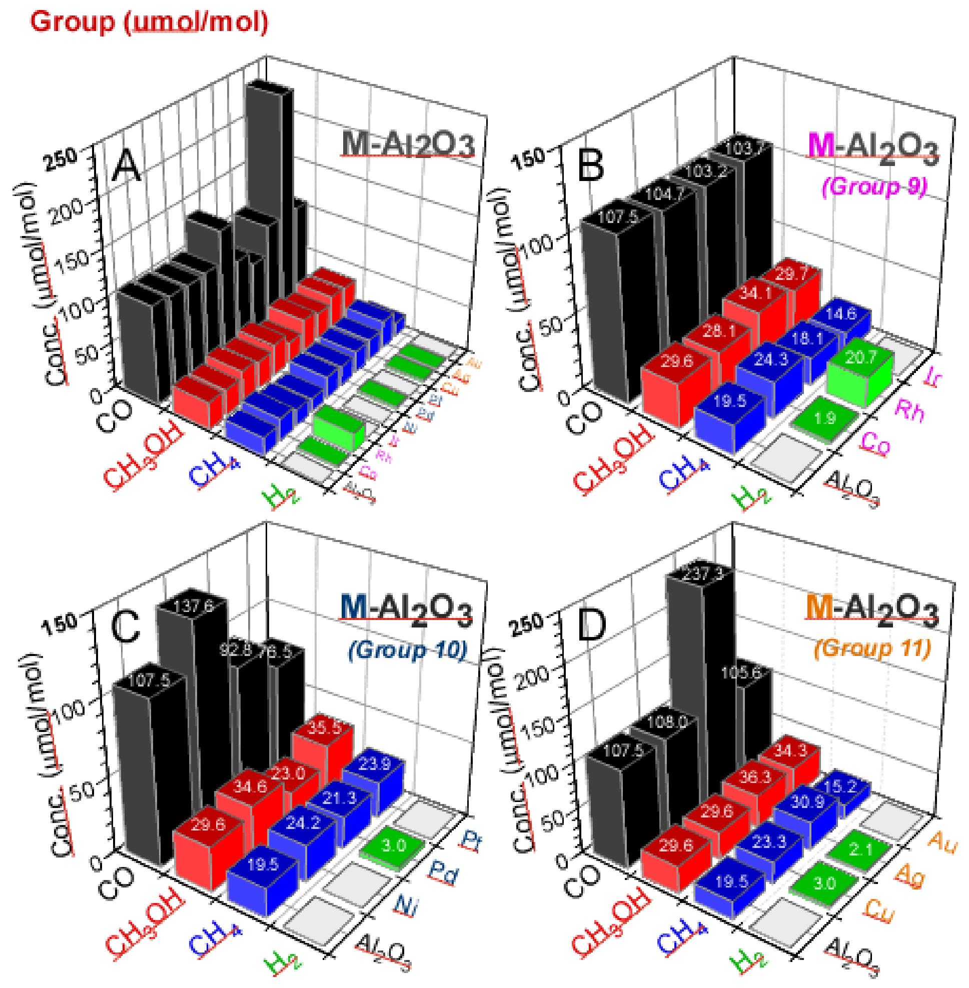

Figure S8. CO_2 reduction CO, CH_4, and CH_3OH yields (μmol/mol) over bare and M-loaded Al_2O_3 nanosheets, group 9: (Co, Rh and Ir)-Al_2O_3, group 10: (Ni, Pd and Pt)-Al_2O_3, and group 11: (Ir, Pt and Au)-Al_2O_3.

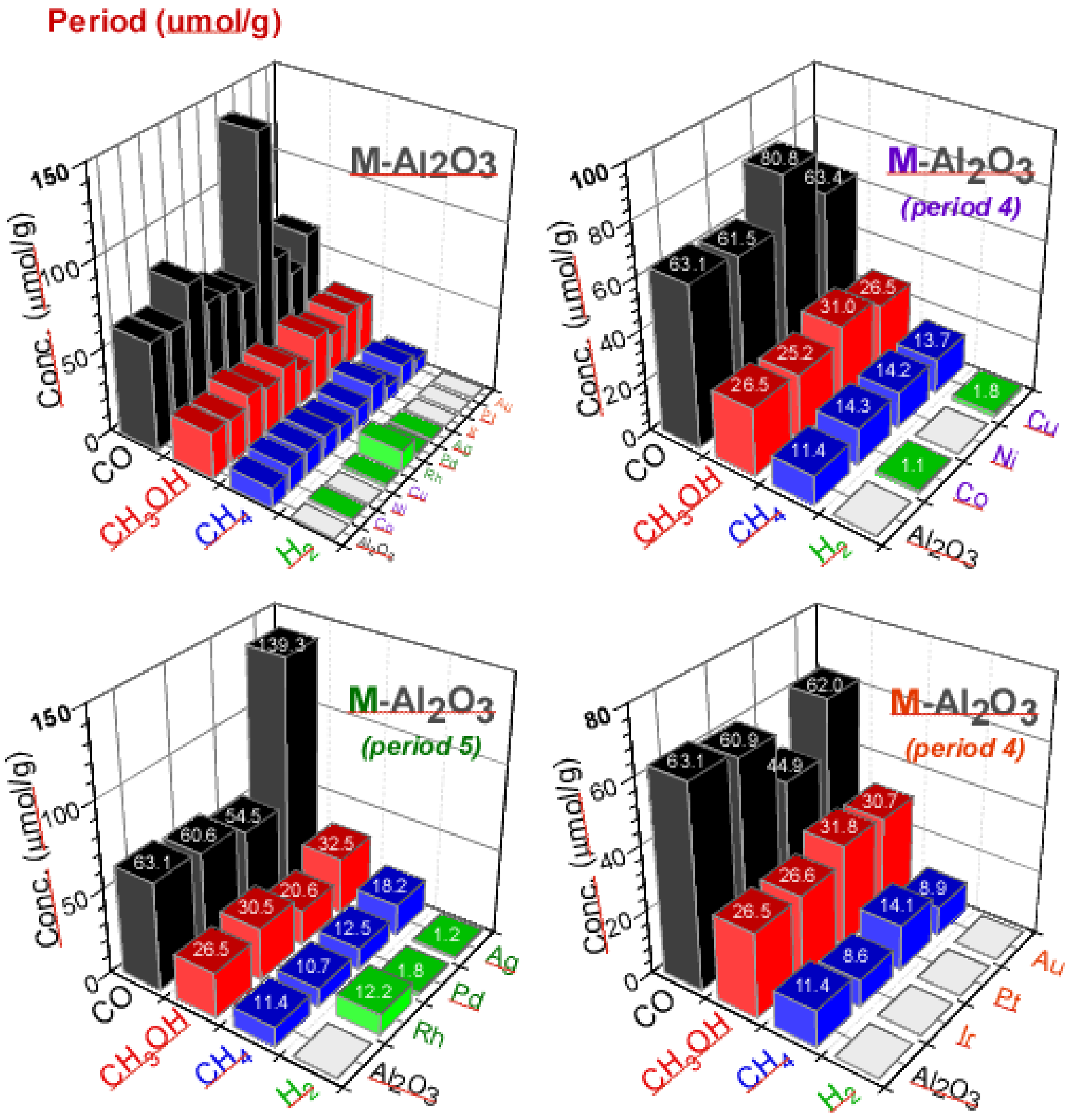

Figure S9. CO, CH_4, and CH_3OH yields (μmol/g) for over bare and M-loaded Al_2O_3 nanosheets, (Co, Ni and Cu)-Al_2O_3, (Rh, Pd and Ag)-Al_2O_3, (Ir, Pt and Au)-Al_2O_3.
